# An early-screening biomarker of endometrial carcinoma: NGAL is associated with epithelio-mesenchymal transition

**DOI:** 10.18632/oncotarget.13340

**Published:** 2016-11-14

**Authors:** Ting Li, Li Yu, Jia Wen, Qinping Liao, Zhaohui Liu

**Affiliations:** ^1^ Department of Obstetrics and Gynecology, Peking University First Hospital, Beijing, China; ^2^ Department of Obstetrics and Gynecology, Tsinghua Changgung Hospital, Beijing, China

**Keywords:** neutrophil gelatinase-associated lipocalin, endometrial cancer, epithelio-mesenchymal transition, epidermal growth Factor

## Abstract

neutrophilgelatinase-associated lipocalin is currently one of the most interesting and enigmatic proteins involved in the development of malignancies. In this study, we found that the expression of neutrophilgelatinase-associated lipocalin was up-regulated in endometrial cancer tissues and cell lines, significantly increased in early-grade ones, suggesting it may serve as a biomarker for early-stage screening for endometrial carcinoma. Moreover, neutrophilgelatinase-associated lipocalin was up-regulated in Ishikawa cells under going epithelio-mesenchymal transition induced by epidermal growth factor (5 ng/ml). Up-regulation of neutrophilgelatinase-associated lipocalin may correlate with the down-regulation of E-cadherin expression, up-regulation of Vimentin expression, enhanced cell migration, invasion and proliferation, which are the typical hallmarks of epithelio-mesenchymal transition processes. neutrophilgelatinase-associated lipocalin may play a dual role during tumorigenetic and developmental processes of endometrial carcinoma. These results suggested neutrophilgelatinase-associated lipocalin to be a potential molecular target in the early diagnosis and treatment of endometrial carcinoma. Further studies are warranted to clarify the molecular mechanisms behind the expression and function of neutrophilgelatinase-associated lipocalin and epithelio-mesenchymal transition.

## INTRODUCTION

Worldwide, Endometrial carcinoma (EC) is one of the commonest malignancies in the female genital tract [1, 2]. Although EC is a highly curable malignancy with the overall 5-year survival more than 80% [3], its specific morbidity is increasing rapidly accompanying longer life expectancy [4], an epidemic of obesity [5], physical inactivity and sedentary behavior [6], diabetes mellitus [7] and arterial hypertension [8].

Neutrophil Gelatinase-Associated Lipocalin (NGAL), a multifunctional 25-kDa extracellular protein, is a new member of the lipocalin superfamily, also referred to as Human Neutrophil Lipocalin(HNL), lipocalin-2 (lcn2), siderocalin or 24p3/uterocalin(the mouse ortholog) [9] and neu-related lipocalin(the rat ortholog)10. The solution structure of NGAL comprises a β-barrel formed by eight β-strands. This β-barrel works as a calyx binding and transporting ions as well as small hydrophobic molecules, including the bacterial iron-trafficking siderophore enterochelin from Gram negative bacteria, bacillibactin from Gram positive. NGAL exerts anti-bacterial function by binding siderophores and their ferric complexes, thereby intercepting the delivery of iron to the bacteria [11]. NGAL has been identified as an early biomarker for prediction of acute kidney injury (AKI), but also shows potential predictive value for clinical outcomes of renal replacement therapy and mortality [12]. NGAL can additionally improve tubule repair caused by AKI by promoting the formation of renal distal tubular and collecting ducts.

A large number of clinical data have confirmed that it also acts as an acute-phase protein with up-regulation not only in different inflammatory conditions, immune response, apoptosis and cell differentiation, but also in the occurrence, development, invasion and metastasis of various human tumors, including thyroid [13], breast [14], colon [15, 16] and ovary [17] carcinomas. On the one hand, this iron regulatory protein can bind to matrix metalloproteinase-9 (MMP-9) to form MMP-9/NGAL complex, protecting MMP-9 from proteolytic degradation commonly associated with the clinical progression of human cancers. On the other hand, NGAL inhibits the cancer-promoting factor HIF-1 alpha and FAK phosphorylation, hindering the synthesis of VEGF, so at this time NGAL serves as a protective factor [18].

In malignant tumor cells, NGAL can promote epithelio-mesenchymal transition (EMT) which may accelerate the malignant invasion and metastasis [19]. NGAL-mediated iron acquisition promotes proliferation and survival of the tumor cells [13]. EMT is characterized by reprogramming and reshaping from epithelial sheets to migratory fibroblast-like cells for locomotion and invasion [20, 21]. The program, considered an important mechanism of tumor invasion and metastasis in many common cancers, allows a polarized epithelial cell undergo multiple biochemical changes that enable it to assume a mesenchymal cell phenotype, occuring during early embryonic development as well as the pathogenesis of cancer [22]. The mutation or loss of PTEN, PIK3CA and KRAS currently happen in most cases of endometrial carcinoma, all of which play an important role in EMT related signaling pathways. In addition, a decline or deficiency of E-cadherin is represented in both type I and type II with the poor prognosis, including myometrium invasion, poor differentiation, lymphatic and stromal infiltration and so on [23–25]. In solid tumors, EMT endows tumor cells with the ability to detach the primary site and migrate into the blood, lymph vessels or exudates; these cells then undergo MET, the reverse process that allows them to form new metastasis at distant target organs, retrieving its previous characteristics like the primary malignancy [22]. All phenomena show that EMT process plays an important role in the development, invasion and metastasis of EC. The EMT process induced by various growth factors, which are closely related to many cell signaling pathways, thus EMT may be one of the important mechanisms of invasion and metastasis of EC.

Thus, the precise cancer-promoting or -supressing function of NGAL has not been well defined with characteristics of species difference and histology difference. In this study, we examined the expression and possible functions of NGAL and relevant EMT in EC.

## RESULTS

### NGAL is a putative early-screening biomarker for EC

**Figure 1:**
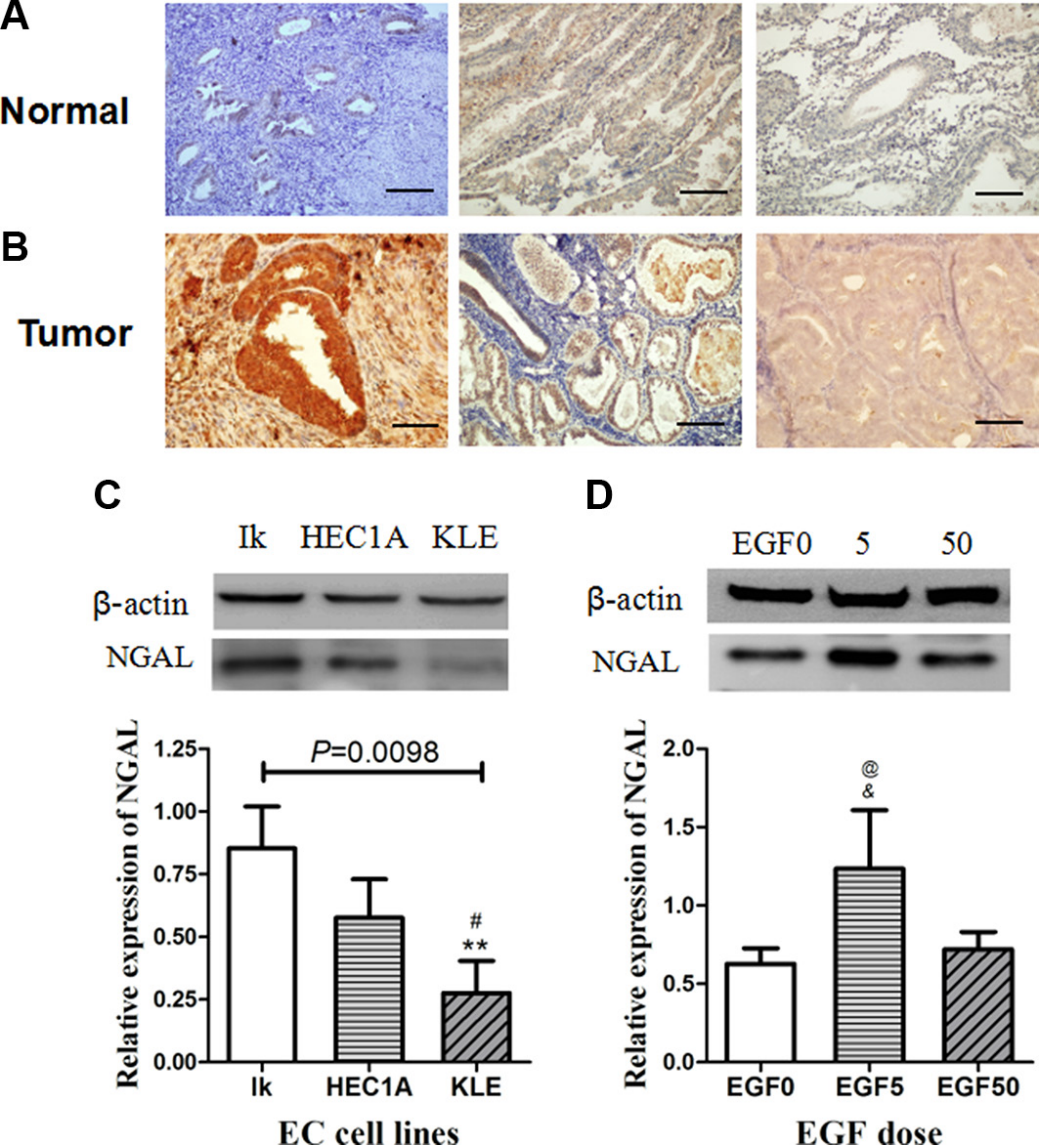
(**A–B**) Immuno-histochemical detection of NGAL expression in endometrial tissues. Photomicrographs of immunostaining in human normal endometrium (A:proliferative phase & early secretory phase) and endometrial carcinoma (B: Grade 1, Grade 2 and Grade 3). Immunoreactivities for NGAL are negligible in normal endometrium and Grade 3 endometrial carcinoma, and strongly positive in Grade 1 & 2 endometrial carcinoma. Representative fields were photographed at ×200 magnification. (**C–D**) Western blot analysis of NGAL expression in endometrial cancer cell lines. (C) and EGF-treated Ishikawa cells (D). The blot was probed for β-actin to ensure equal protein loading. Each sample was performed 3 times. In (C), **: significant difference from Grade 1 (*P* = 0.003); #: significant difference from Grade 2 (*P* = 0.050). In (D), *: significant difference from control (EGF 0) cells (*P* = 0.019); #: significant difference from EGF (50 ng/ml)-treated cells (*P* = 0.035). The error bars indicated standard deviation.

We measured the expression levels of NGAL protein in 41 archived EC samples (3 atypical hyperplasia with malignant development, 14 Grade 1, 18 Grade 2 and 6 Grade 3), and 23 normal endometria samples using Western blot assays. Western blot analysis showed the mean relative NGAL protein level of EC was significantly higher than that of normal control (0.739 ± 0.092 vs. 0.222 ± 0.039, *P* < 0.0001), with the highest level in Grade 1 EC which was 4.1-fold higher than control samples (0.913 ± 0.156 vs. 0.222 ± 0.039; Table 1). Immunohistochemical Staining indicated that NGAL immunoreactivity was not evident in normal endometrial glands or stroma nor the myometrium in proliferative phase and secretory phase (Figure 1A). Low-grade EC tissue showed consistent NGAL positivity of moderate to strong degree, mainly localized in the gland within the cytoplasm and the cell surface membrane, and the strongest staining intensity was seen in Grade 1 tumor; and the expression of NGAL in high-grade poorly differentiated EC tissues was negative (Figure 1B).

**Table 1:** Correlation between the clinicopathologic characteristics and expression of NGAL protein in normal endometrium and EC

Characteristics	*N*	Relative NGAL expression	*P* value
**Age(y)**			
** ≤ 50**	32	0.38 ± 0.06	0.036*
** > 50**	32	0.72 ± 0.12	
**Group**			
** Normal**	23	0.22 ± 0.04	< 0.0001*
** Malignant**	41	0.74 ± 0.09	
**Histologic type**			
** Type I**	36	0.76 ± 0.10	0.627
** Type II**	5	0.62 ± 0.16	
**FIGO stage**			
** I**	33	0.77 ± 0.11	0.692
** II**	2	0.43 ± 0.09	
** III**	6	0.66 ± 0.19	
**Histological grade**			
** Normal**	23	0.22 ± 0.04	< 0.0001*
**Atypical hyperplasia**	3	0.42 ± 0.05	
** Grade 1**	14	0.91 ± 0.16	
** Grade 2**	18	0.73 ± 0.16	
** Grade 3**	6	0.51 ± 0.14	
**Depth of myometrial invasion**			
** < 50%**	58	0.72 ± 0.09	0.689
** ≥ 50%**	19	0.80 ± 0.25	
**Lymph node metastases**			
** Negative**	37	0.74 ± 0.10	0.913
** Positive**	4	0.77 ± 0.28	
**Serum CA125†**			
** Negative**	15	1.12 ± 0.18	0.002*
** Positive**	10	0.45 ± 0.11	

*P < 0.05(Independent-Sample *t* test or Mann-Whitney *U* test; one-way ANOVA or Kruskal-Wallis *H* test).

^†^Some of the EC patients have not detected serum CA125.

Of the cell lines tested, Type I endometrial cancer cell lines Ishikawa (Grade 1) and HEC-1A (Grade 2) expressed NGAL, while Type II endometrial cancer cell lines KLE (Grade 3) showing weak expression of NGAL. The expression pattern of NGAL in cell lines shared similar expression pattern with the histological results of human tissue. The relative expression for NGAL in the Ishikawa cells, Hec-1-A cells and KLE cells was 0.852 ± 0.167, 0.576 ± 0.154 and 0.276 ± 0.128 (mean ± SD, *F* = 11.037, *P* = 0.0098), respectively (Figure 1C). The expression of NGAL decreased in a stepwise manner with advance in grading of tumor.

### EGF-induced EMT cell morphological changes

**Figure 2:**
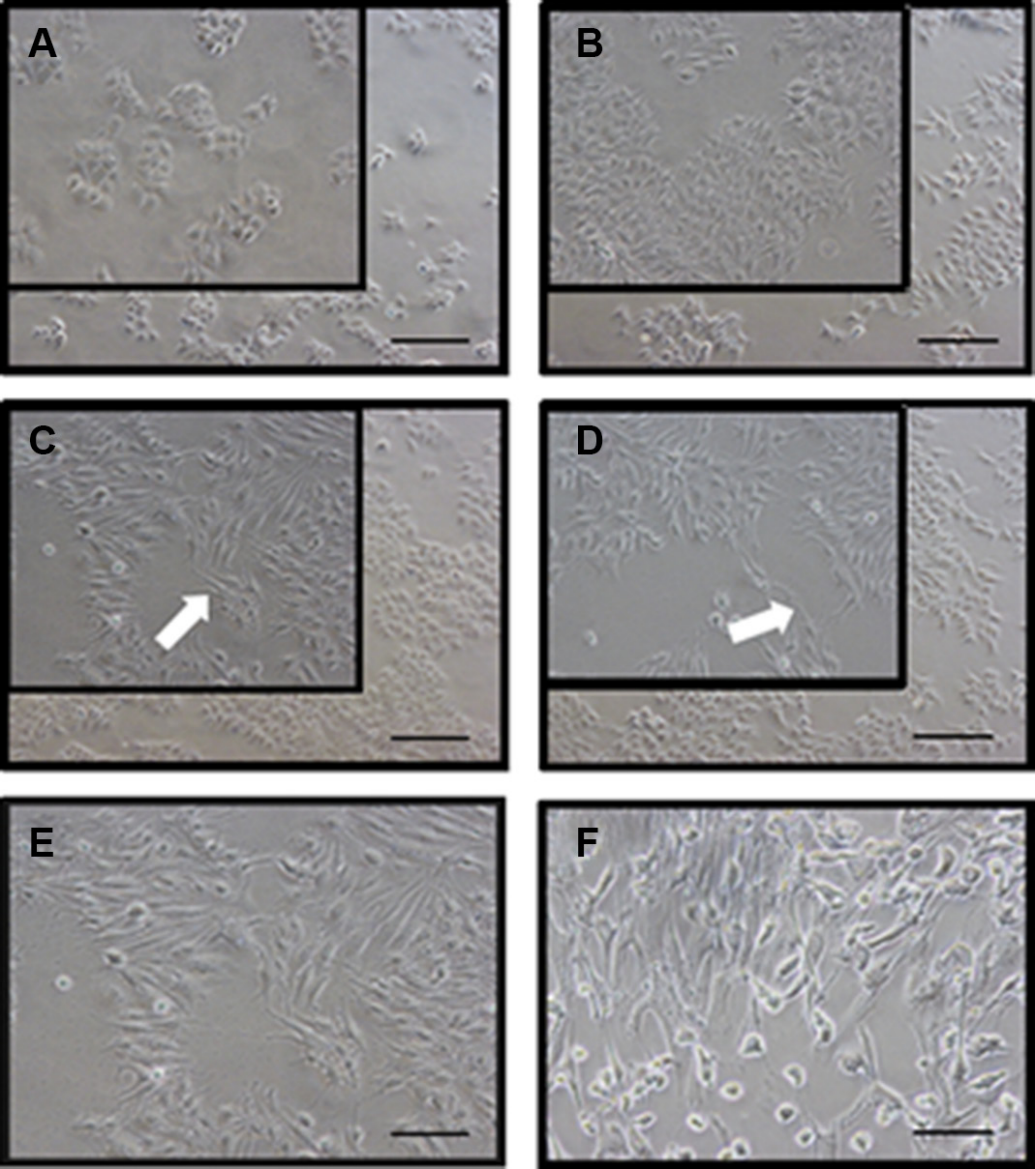
EGF induces a typical EMT morphological changes in HEC-1A cells (**A**) represents control cells at 0 h; (**B**) represents control cells after 48 h; (**C**) represents HEC-1A cells induced by EGF 5 ng/ml for 48 h and (**D**) represents that by EGF 50 ng/ml for 48 h. Pseudopodia (white arrows) were noted in the spindle-shaped cells induced by EGF-mediated EMT. The EMT cells induced by EGF 50 ng/ml for 48 h (**E**) showed similar morphology with primary human fibroblast cells (spindle-like cells, **F**). Representative fields were photographed at ×200 magnification.

EMT-like features can be induced in cells by treatment with substances such as EGF, TGF β 1 or HGF17 26. The HEC-1A control cells, with a “paving stone” glandular epithelium cell shape, merely branched filopodia-like protrusions, dendritic pseudopodia. After been treated with 5 and 50ng/ml EGF for 48h, the cells dramatically changed their morphology to “spindle” fibroblast-like shape in a dose-dependent manner, accompanied by the looser cell-cell connection and the longer filopodia-like protrusions and pseudopodia (Figure 2).

### EGF induced EMT changes of cell phenotype

**Figure 3:**
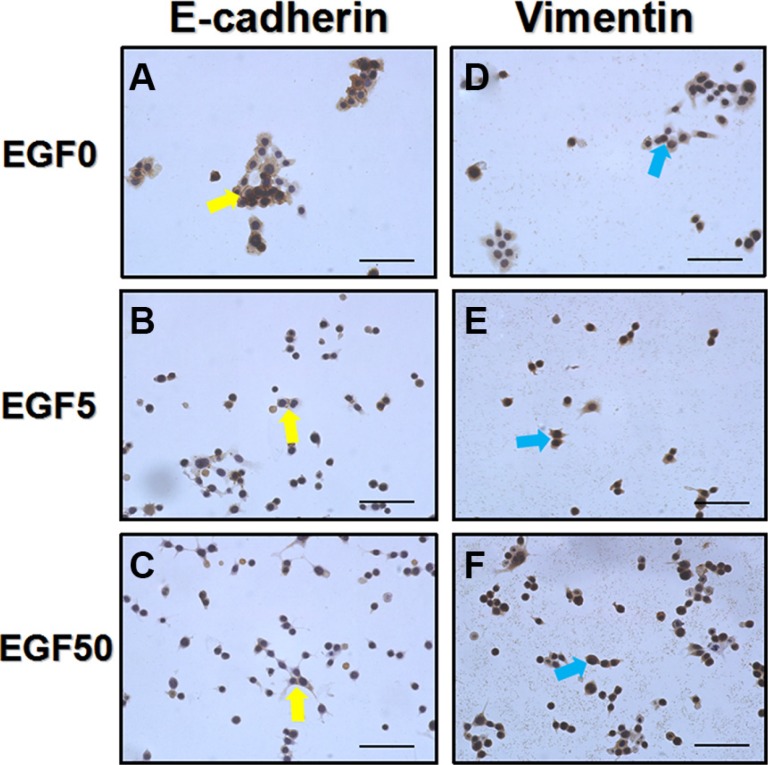
EGF induces a typical EMT cell phenotype changes in Ishikawa cells (**A–C**) E-cadherin expressions (yellow arrows) after the treatment with EGF 0 ng/ml, 5 ng/ml and 50 ng/ml for 48 h, respectively. (**D–F**) Vimentin expressions (blue arrows) after the treatment with EGF 0 ng/ml, 5 ng/ml and 50 ng/ml for 48 h, respectively. EGF treated Ishikawa cells showed a low expression of E-cadherin and high expression of vimentin. Representative fields were photographed at ×200 magnification.

Concomitant with the morphology results, cell phenotypes were also changed as the process of EMT (Figure 3). In both Ishikawa and HEC-1A cell lines, the EGF treatment could significantly up-regulate typical mesenchymal marker vimentin and down-regulate epithelial marker E-cadherin expression compared to control, indicating the cells had indeed undergone EMT, which is in agreement with a previous report [27]. Interestingly, like the changes in morphology, these EMT alterations of cell phenotypes induced by EGF signaling were dose-dependent.

### Different degrees of EMT modulate NGAL expressions in EC

The above- mentioned results have confirmed that EGF, a known EMT-inducer in other epithelial malignancies [17, 28], could induce dose-dependent changes of EMT in Ishikawa and HEC-1A cell lines. 5ng/ml and 50 ng/ml EGF induced a relative low degree and high degree of EMT, respectively. We found that NGAL expression in low degree of EMT (EGF 5 ng/ml) group was significantly higher than that of the control group (*P* = 0.019) and high degree of EMT (EGF 50 ng/ml) group (*P* = 0.035), while the comparison between control group and high degree of EMT (EGF 50 ng/ml) group was not significantly different (*P* = 0.646; Figure 1D). These results indicate that NGAL expression is up-regulated in the transition of EC epithelial cells to mesenchyme-like cells(EGF 5 ng/ml) but lack of NGAL expression in mesenchyme-like cells (EGF 50 ng/ml) shown in Figure 1D, so only EGF 5 ng/ml were used in further study.

### EMT enhances the motile function of EC cells

**Figure 4:**
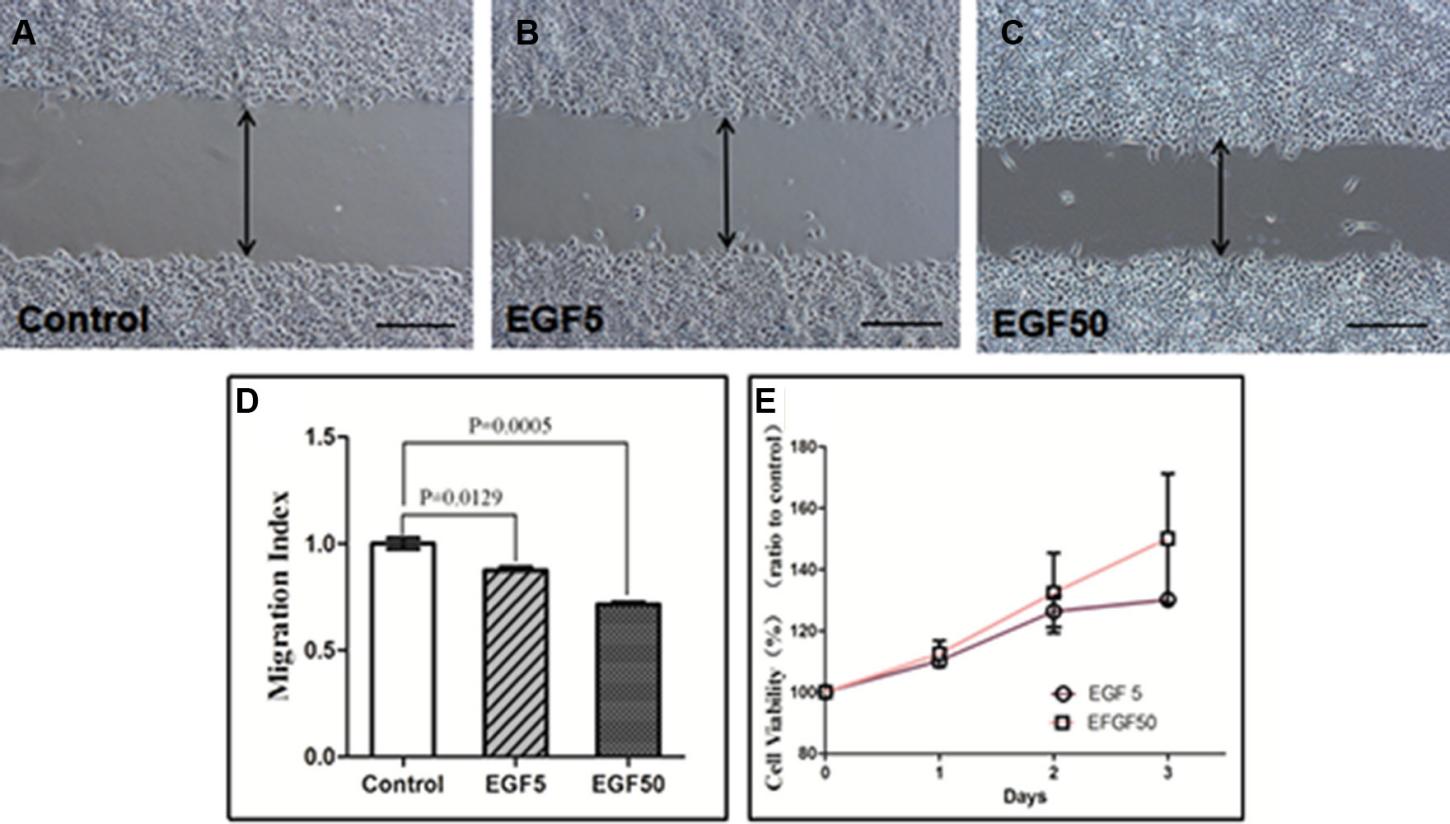
Wounding assay in EGF-treated Ishikawa cells (**A**) Control cells, (**B**) EGF-treated(5 ng/ml) cells, (**C**) EGF (50 ng/ml) -treated cells over 24 h. Representative fields were photographed at ×100 magnification. (**D**) The Migration Index of cells treated with 5 ng/ml and 50 ng/ml EGF were significantly different from that of untreated control (*P* = 0.0129 and *P* = 0.0005, respectively). (**E**) CCK-8 assay in EGF-treated Ishikawa cells. Values are given as mean ± standard deviation. The experiment was performed 3 times in duplicate.

**Figure 5:**
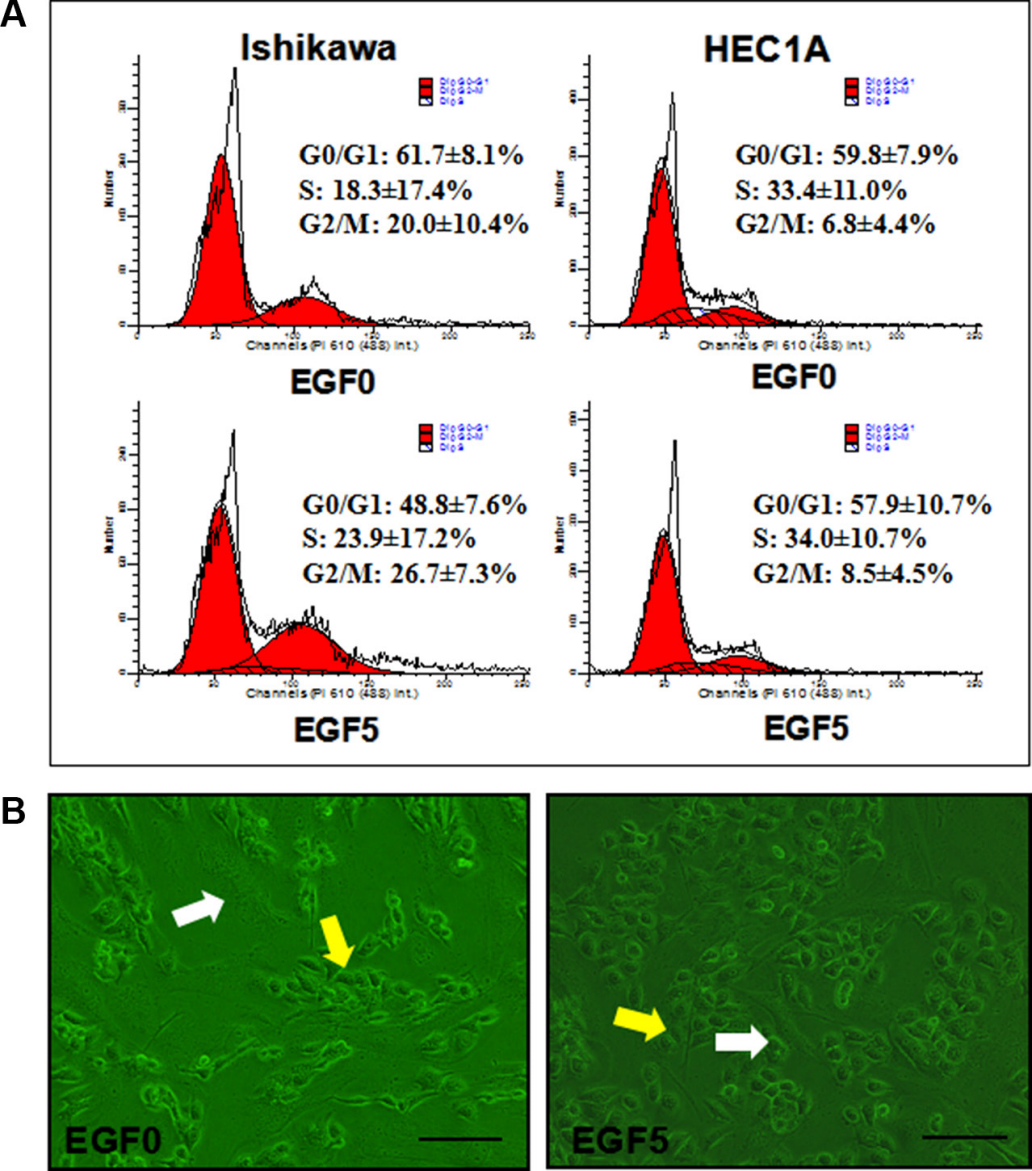
**(A)** Effect of EGF(5 ng/ml)-induced EMT on cell cycle distribution in Ishikawa and HEC-1A cell lines. The experiment was performed 3 times in duplicate. (**B**) Monolayer-invasion assay. Ishikawa cells (yellow arrows) were added to the confluent fibroblast monolayer (white arrows). Representative fields were photographed at ×200 magnification.

To further elucidate the role of NGAL in endometrial tumor progression and motile function, Ishikawa cells were induced to undergo EMT in the presence of EGF. A wounding assay was used to determine the motile function of cells via estimating their ability to migrate into an artificially produced wound (Figure 4). Cell migration was determined by Migration Index (MI), calculated by dividing the distance of wound in EGF-treated cells by that in control cells. EGF promoted wound repair of Ishikawa cells considerably by 24h. Wounding assay results showed that the MI of control group was 1.000 ± 0.044, of EGF-treated (5 ng/ml) cells was 0.874 ± 0.026, and of EGF-treated (50 ng/ml) cells was 0.716 ± 0.017 (*F* = 63.15, *P* < 0.0001). As shown in Figure 5, 5 ng/ml EGF-treated cells and 50ng/ml EGF-treated cells migrated significantly faster than the control group (*P* = 0.0129, *P* = 0.0005), while no closure of the wound was observed.

### EMT promotes the proliferation of EC cells

To further evaluate the effect of EGF-induced EMT upon EC proliferation, we then performed flow cytometry (Figure 6) and CCK-8 assay. The results showed that EGF (5 ng/ml) significantly increased the proportion of cells in S phase and G2/M phase while significantly decreasing the proportion of cells in G0/Gl phase. Compared with control cells, the mean SPF was increased by 5.56%, the mean PI of 50.93 ± 7.53% was significantly higher than that of untreated cells 38.34 ± 8.13% (*P* = 0.006). However, EGF exerted a weak proliferation effect on HEC-1A with the mean SPF was increased by merely 0.63% and the mean PI of 42.18 ± 9.30% was still higher than untreated cells 40.19 ± 7.81% (*P* = 0.205). These results suggest that EGF-induced EMT could promote the cell cycle of EC and DNA synthesis via increasing the proportion of S phase and G2/M phase (Figure 5).

Consistent with enhanced cell cycle, EGF (5 ng/ml and 50 ng/ml) treatment also increased cell growth compared to control untreated cells after 24 h, 48 h and 72 h (Figure 4E). 24 h: The cell viability of EGF-treated (5 ng/ml) cells (*P* = 0.002) and EGF-treated (50 ng/ml) cells (*P* = 0.036) was significantly higher than that of control. 48 h: The cell viability of EGF-treated (5 ng/ml) cells (*P* = 0.013) and EGF-treated (50 ng/ml) cells (*P* = 0.050) was significantly higher than that of control. 72 h: The cell viability of EGF-treated (5 ng/ml) cells (*P* < 0.0001) and EGF-treated (50 ng/ml) cells (*P* = 0.054) was significantly higher than that of control.

### EMT promotes the invasion of EC cells

To investigate the potential influence of EGF-induced EMT on EC cell invasion, we used a direct co-culture approach allowing direct contact between Ishikawa cells and primary human fibroblast monolayer which imitates normal stroma(Figure 5B). After 24 h the number of Ishikawa cells in each field that had migrated into the fibroblast monolayer was determined and used as an indicator of invasive capacity. The infiltration of the EGF-treated (5 ng/ml) cells into the astrocyte monolayer was significantly greater than untreated cells (*P* = 0.003). These data (unshown) suggest that EGF-induced EMT dramatically enhances the invasive behavior of EC cells.

## DISCUSSION

Our previous study have revealed that the expression of NGAL was increased in neoplastic endometria especially in early grade endometiral tumors, while lack in proliferative and secretory phases of normal endometria, suggesting that the protein is oncogenic [29] (Figure 1). These results are in accordance with Miyamoto's first report of NGAL in EC [30, 31]. The present study verified a significantly increased NGAL expression in early-grade Ishikawa EC cell line when compared to high-grade HEC-1A and KLE cell lines, suggesting it may serve as a biomarker for early-stage screening for EC. NGAL expresses mostly as the monomeric form in tumor cell lines, and mainly in the well-diferentiated glands in Grade 1 and 2 tumors, while week immunoreativity observed in high-grade tumors [32]. NGAL has been reported to promote early events of metastasis in many tumors. The potent tumor-promoting effects of NGAL can be explained by stabilizing gelatinase B (MMP-9) and inducing EMT in several malignant neoplasms, thereby enhancing the invasion ability [33, 34]. However on the other hand, NGAL also plays its tumor-suppressing effects as an epithelial inducer in malignancy and a suppressor of metastasis [35] and, Surprisingly, NGAL yet is able to inhibit EMT in hepatocellular carcinoma [36] and pancreatic cancer [37]. The different relationship between NGAL and EMT are still controversially discussed. We point out that NGAL expression and functions have showed huge differences between the organizations and species. This duplicity in function highlights NGAL and its potential signaling pathways may be the key targets for cancer therapy [38].

Cancer progression is a multistep process during which cells undergo alterations of their normal functions and culminate into the highly malignant and metastatic phenotype that progressively lead to the carcinogenesis [13]. Given that EC often arises from endometrial glandular cells [30] and NGAL is overexpressed in these epithelial cells rather than stroma cells, we postulate that NGAL may work as a critical factor during oncogenesis. The mechanisms of modulating NGAL expression are not fully understood. Howbeit Hanai et al [39] and Lim et al [17] have successively proposed that NGAL expression is up-regulated in the epithelial cells which are thought to be the source of most breast or ovarian carcinomas, however, NGAL seems to be associated with the epithelial phenotype of malignancy, gradually losing as epithelial cancers progress and become undifferentiated.

Since NGAL is a putative epithelial inducer of EC, we further investigate the modulation of NGAL in EMT, a key event in the tumor invasion process, and the role of EMT status in EC cells. In the present study, we verified that EGF can also induce the mesenchymal changes in Ishikawa and HEC-1A cell lines characterized by loss of epithelial traits and the acquisition of mesenchymal characteristics, which meet the proposed four functional criteria of EMT at the first Boden International Conference on EMT in Port Douglas, Australia, 2003: (1) loss of cell polarity; (2) Spindle-like or fibroblast-like elongate morphology; (3) filopodia formation; and (4) invasive motility [40].

EGF can induce EMT in a variety of tumor cells including breast cancer cell (EGF 50 ng/ml) [41], ovarian cancer and (EGF 10 ng/ml) [17], cervical cancer cell (EGF 50 ng/ml) [42]. A hallmark of EMT is the loss of E-cadherin expression and the up-regulation of Vimentin. It is noteworthy that these EMT alterations of morphology and cell phenotypes in EC cells induced by EGF signaling were dose-dependent, however, only the dose of 5 ng/ml could trigger the up-regulation of NGAL expression. We conclude that NGAL expression is up-regulated by EGF (5 ng/ml)-induced EMT accompanying by the enhancements in the migration, invasiveness, proliferation of EC cells, partially through morphologic and molecular changes. EGF binding or stimulating EGF receptor triggers numerous downstream signal transduction pathways such as PI3K/AKT/ERK43 that ultimately lead to EMT-induced cellular responses and the expression of NGAL. In SKOV3 and OVCA433 cell lines, NGAL, known as an acute phase protein, has been shown to be stimulated by EGF (10 ng/ml) treatment after 24 h but diminished after 48 h [17]. Moreover, Tong et al acclaimed NGAL was down-regulated significantly after EGF treatment along with a concomitant reduction of E-cadherin in pancreatic ductal adenocarcinoma [44]. The discrepancies may be due to the duplicity in function of NGAL varying from tissue to tissue. One of our unpublished demonstrated that exogenous recombinant NGAL exerts little effects on morphology and EMT-related phenotypes in Ishikawa and HEC-1A cells. Further studies should focus on the effects of endogenous NGAL. To our knowledge, the research on the effect of EGF-induced EMT on EC cells has not been reported, and this is the first report of the effect of EGF-induced EMT on EC cells and NGAL expression. No matter the duplicity in function of NGAL, these results suggest NGAL to be a novel target in the early diagnosis and treatment of EC.

EGF-induced EMT in EC cells involves the coordination of multiple signaling pathways. The interaction between NGAL and EMT is still under intense investigation. There is a lot that we do not understand about NGAL and EMT in EC. The biggest weakness in our understanding is the connection between NGAL expression and cell behavior. Research regarding the function of NGAL and the relationship with EMT in EC is in its initial stages, and many phenomena need to be investigated. Further studies should explore the effects of endogenous NGAL.

## MATERIALS AND METHODS

### Ethical approval of the study protocol

Written informed consent was obtained from all donors. All experimental procedures and protocols were approved by the Ethics Committee of Peking University First Hospital (Beijing, China).

### SDS-PAGE and Western blot analysis

From 2015 to 2016, of the 64 tissues studied by Western blot analysis, 41 archived EC samples (3 atypical hyperplasia with malignant development, 14 Grade 1, 18 Grade 2 and 6 Grade 3), and 23 normal endometria samples were obtained in Peking University First Hospital, Beijing, China. In the 41 EC cases, the median age of the patients was 54 years (range from 40 to 76).

The endometrial cancer cell lines Ishikawa (Type I, Grade1), HEC-1A (Type I, Grade2) and KLE (Type II, Grade3) were cultured with the routine method. Tissue and cultured cells were subjected to Western blot analysis were done as previously described [30]. Briefly, tissue and cell lysate were analyzed in 12% SDS-PAGE. The protein samples were separated by electrophoresis and transferred from the gel to nitrocellulose membranes. Blocking was performed with 5% nonfat milk for 1 hour at room temperature, then stained indirectly with primary anti-NGAL antibody at 4°C overnight and then incubated with HRP-conjugated secondary antibody. Bound secondary antibodies were visualized using enhanced chemi-luminescence (Pierce).

### Cell culture and EGF treatment

The endometrial cancer cell lines were cultured at 37°C in DMEM/F12 medium supplemented with 10% FBS. The cells were treated with recombinant human EGF (Peprotech, RockyHill, NJ, USA) incubated in low FBS free medium at concentrations of 5 ng/ml and 50 ng/ml. The control group was treated as described above, but EGF was replaced by 0.01 M phosphate-buffered saline.

### Immunohistochemistry on cell lines and tissue samples

Cells were cultured as described above, then fixed in 4% formaldehyde for 20 min and permeabilized with 0.3% Triton X-100 for 30 min. Three cases of normal endometrium(1: proliferative phase, 2: secretory phase) and three human endometrial carcinoma tissues of various differentiation grades for the comparison of the normal tissues, Grade 1, Grade 2 and Grade 3 tumors were obtained from Peking University First Hospital. The pathology diagnosis and tumor grade were determined by 2 staff pathologists in the Department of Pathology, Peking University First Hospital. We have retrospectively measured the expression level of NGAL in seventy-seven cases with endometrial carcinoma and twenty-eight cases with normal endometrium by western bloting [29]. Human endometrial cancer tissues were fixed in 10% formalin, embedded in paraffin, and sectioned. Endogenous peroxidase were blocked in 3% H_2_O_2_ for 10 min then sections were incubated with the primary rabbit antibody (anti-NGAL antibody, anti-E-Cadherin 1:100 and anti-Vimentin 1:100) following the avidin-biotin-peroxidase procedure using an EnVision Automated Immunostainer (Dako). Nuclei were stained with hematoxylin-eosin (H&E) for routine microscopy. The slides were examined under a light microscope (AH3-RFCA; Olympus, Tokyo, Japan).

### Wound healing assay

Wound Healing Assay was performed to assess directional cell migration *in vitro*. Ishikawa and Hec-1-A cells were cultured in 6-well plates until yielding 90% confluent monolayers for wounding, then starved with FBS-free medium and incubated for 24 hours. A P200 micropipette tip was used to manually make a “wound” in a cell monolayer, capturing the images at 0 h and 24 h after wounding. Five representative fields were marked and measured. Results were expressed as a migration index, that is, the distance migrated by EGF treated relative to the distance migrated by the control group.

### Cell proliferation assay

CCK-8 (Dojindo Laboratories, Tokyo, Japan) was used to evaluate cell viability *in vitro*. Hec-1-A cells were cultured in 96-well plates in a humidified atmosphere containing 5% CO_2_ at 37°C for 24 h. After washing twice with PBS, cells in each well were incubated in 100 μl DMEM/F12 containing 10 μl CCK-8 reagent at 37°C for 1 h. The absorbance at 450 nm (A_450_) was measured using a Muiltiskan GO micro-plate. Results were expressed as cell viability.

Cell viability (%) = [A_450_ (treated)- A_450_ (blank)]/[A_450_ (control)- A_450_(blank)] × 100%

### Flow cytometry analysis of cell cycle

Hec-1-A cells were starvested by serum-free medium for 24h, then collected and fixed in 70% cold ethanol for 4°C overnight. After incubation with 100 μl RNaseA (50 μg/ml) 37°C for 30 min, the cells were stained with 400 μl propidium iodide (PI) at 4°C for 30min. For each sample, a minimum of 20,000cells were collected and counted for Flow cytometry analysis, then calculated proliferative index (PI) and S-phase fraction (SPF), as follows. Experiments were performed in triplicate independently.

$$\text{PI}=\frac{S+G2/M}{G0/G1+S+G2/M}\times 100\%\text{ SPF}=\frac{S}{G0/G1+S+G2/M}\times 100\%$$

### Monolayer invasion assay

The human fibroblasts were seeded 6-well plates until confluent. The single-cell suspension of 1 × 10^5^/ml of the Ishikawa cells were carefully added on the confluent fibroblast monolayer, ensuring the Ishikawa cells were in direct contact with fibroblasts. The wells were washed three times to remove all non-penetrating cells. After 24 h of incubation, penetrated Ishikawa cells were photographed using a phase contrast microscope and counted in 5 fields of view at × 200 magnification. The number of penetrated Ishikawa cells were counted and photographed. Experiments were performed in triplicate independently.

### Statistical analysis

All the experimental data were expressed as Mean ± SEM and analyzed by Student's *t*-test or one-way ANOVA using SPSS 13.0 statistical software. *P* < 0.05 was considered statistically significant.
